# Large Fermi‐Energy Shift and Suppression of Trivial Surface States in NbP Weyl Semimetal Thin Films

**DOI:** 10.1002/adma.202008634

**Published:** 2021-05-04

**Authors:** Amilcar Bedoya‐Pinto, Defa Liu, Hengxin Tan, Avanindra Kumar Pandeya, Kai Chang, Jibo Zhang, Stuart S. P. Parkin

**Affiliations:** ^1^ Max Planck‐Institute of Microstructure Physics Weinberg 2 Halle (Saale) 06120 Germany

**Keywords:** Fermi arcs, Fermi‐level engineering, topological surface states, Weyl semimetal thin films

## Abstract

Weyl semimetals, a class of 3D topological materials, exhibit a unique electronic structure featuring linear band crossings and disjoint surface states (Fermi‐arcs). While first demonstrations of topologically driven phenomena have been realized in bulk crystals, efficient routes to control the electronic structure have remained largely unexplored. Here, a dramatic modification of the electronic structure in epitaxially grown NbP Weyl semimetal thin films is reported, using in situ surface engineering and chemical doping strategies that do not alter the NbP lattice structure and symmetry, retaining its topological nature. Through the preparation of a dangling‐bond‐free, P‐terminated surface which manifests in a surface reconstruction, all the well‐known trivial surface states of NbP are fully suppressed, resulting in a purely topological Fermi‐arc dispersion. In addition, a substantial Fermi‐energy shift from −0.2 to 0.3 eV across the Weyl points is achieved by surface chemical doping, unlocking access to the hitherto unexplored n‐type region of the Weyl spectrum. These findings constitute a milestone toward surface‐state and Fermi‐level engineering of topological bands in Weyl semimetals, and, while there are still challenges in minimizing doping‐driven disorder and grain boundary density in the films, they do represent a major advance to realize device heterostructures based on Weyl physics.

## Graphical abstract

Topological materials host both topological (TP) and trivial (TR) surface states in their band structure, the latter being undesired for topology‐driven applications. A full suppression of trivial surface states is achieved by surface reconstruction of epitaxial NbP Weyl semimetal thin films. Moreover, large shifts of the chemical potential are induced by doping, enabling Fermi‐level engineering of purely topological states.

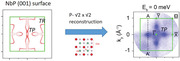

## 1 Introduction

Weyl semimetals (WSMs) are found in both inversion‐symmetry breaking crystals,^[^ ^1^, ^2^, ^3^, ^4^, ^5^ ^]^ such as transition metal monopnictides (Nb, Ta / P, As) of interest here, as well as in time‐reversal symmetry breaking compounds.^[^ ^6^, ^7^, ^8^ ^]^ Early on, most of the exploratory studies focused on assessing the special features of WSMs by direct probing of the electronic structure such as via angle‐resolved photoemission^[^ ^9^, ^10^, ^11^ ^]^ and scanning tunneling spectroscopy.^[^ ^12^, ^13^, ^14^ ^]^ Recently, interest has focused on the exploitation of the unusual properties of their electronic structure to observe unique physical phenomena, such as the chiral^[^ ^15^, ^16^, ^17^ ^]^ and axial‐gravitational anomaly,^[^ ^18^ ^]^ the circular photogalvanic effect,^[^ ^19^, ^20^ ^]^ chiral sound waves,^[^ ^21^, ^22^ ^]^ the surface‐state enhanced Edelstein effect^[^ ^23^ ^]^ or the recently proposed chiral Hall‐effect.^[^ ^24^ ^]^ The observation of most of these effects depends on whether the topological electronic states of the WSMs can be readily accessed. In this regard, the ability to suppress non‐topological (trivial) surface states, as well as to modify the Fermi‐level position to get a desired Fermi surface topology, would allow full access to unveil the role of topological surface states on physical observables, and, in addition, to construct on‐demand Fermi‐surfaces to harness electrical, acoustic or optical measurable outputs. So far, the diversity of electronic structures was achieved through exploring different WSMs, but a genuine control of the shape and size of topological bands in the same material has remained elusive, mostly due to the lack of bottom‐up, ultrahigh‐vacuum synthesis methods that allow for control of the surface termination and Fermi‐level position, for instance by doping or strain. This challenge needs to be overcome to achieve Fermi‐level engineered Weyl semimetal heterostructures, leading to a plethora of novel platforms to explore both fundamental phenomena and device applications based on topology.

In this work, we show two striking modifications of the electronic structure of the type‐I Weyl semimetal NbP, that become accessible due to a successful epitaxial thin film growth synthesis route.^[^ ^25^ ^]^ First, a full suppression of the bowtie‐like (trivial) surface states of NbP is obtained due to the saturation of surface dangling bonds by an ordered phosphorous termination, that manifests itself in a (√2 × √2) surface reconstruction. Second, by chemically doping the surface with Se‐atoms, the Fermi‐energy undergoes a substantial shift of around +0.3 eV (electron doping) while preserving the pristine NbP bandstructure features, thereby enabling the first experimental visualization of the topological band dispersion well above the Weyl points, and highlighting the large Fermi‐level tunability that can be achieved by surface chemical doping in a molecular beam epitaxy process. Our work opens up the possibility of realizing recent theoretical proposals, such as a Weyl semimetal field effect transistor (WEYLFET) that relies on purely topological surface states to achieve a negative refraction via gate‐induced Fermi‐arc tilting,^[^ ^26^ ^]^ or a p–n–p Weyl semimetal junction to induce a large chirality splitting and quantify the Berry phase through electron transport experiments.^[^ ^27^ ^]^

## 2 Results and Discussion

**Figure 1:**
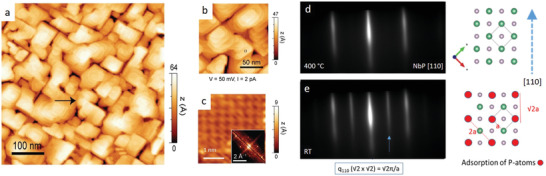
Growth‐induced surface reconstruction in NbP epitaxial thin films. a) Overview scanning tunneling microscopy image of a 15 nm‐thick NbP film (*V* = 10 mV, *I* = 10 pA), revealing rectangular grains between 50 and 100 nm in width and aligned 90° with respect to each other, consistent with the NbP crystal symmetry. b) Magnified (150 nm × 150 nm) topography image (*V* = 50 mV, *I* = 2 pA), featuring atomic terraces along the grains. c) Atomically resolved STM image inside a grain plateau, where a √2 × √2 surface reconstruction of the NbP (001) square lattice plane can be visualized (a derived FFT pattern is shown as inset). d,e) In situ RHEED characterization of the NbP surface at 400 °C (end of the film growth) and after cooling down to room‐temperature in a phosphorous atmosphere, the latter consistent with a phosphorous‐induced √2 × √2 surface reconstruction. e) Adsorption model of phosphorous atoms compatible with a √2 × √2 surface reconstruction.

A detailed characterization of the film surface is essential to understand the process of surface reconstruction and chemical doping. The topography and surface structure of a 15 nm‐thick NbP film is investigated by scanning tunneling microscopy (**Figure** **1**a–c). Square‐ and rectangular‐shaped grains are identified with two preferred orientations (90° rotation) consistent with the in‐plane crystal symmetry of NbP, as shown in Figure 1a. A closer look at the grain topography (Figure 1b) reveals the presence of atomically flat sub‐terraces. The height of each step terrace amounts to 2.8 Å, corresponding to ¼ of the unit cell, i.e., a single Nb–P monolayer. (The step profile along with a structural model of the NbP unit cell along the growth direction (001) are given in Figure S1 in the Supporting Information). Since we rarely find slight deviations of exact unit cell fractions and from the topography statistics, a predominant single surface termination (either Nb or P) is likely throughout the film surface. Most importantly, the atomically resolved topography image (Figure 1c) is characteristic of a square lattice plane with an interatomic distance close to 5 Å, consistent with a (√2 × √2) reconstruction (a structural rearrangement of the surface atoms with periodicity √2 times the NbP lattice constant). The derived fast‐Fourier‐transform (FFT) is shown as inset of Figure 1c. This surface reconstruction occurs during the cooling of the film from 400 °C (during growth) to room‐temperature under a P‐rich atmosphere, as observed by the emergence of additional RHEED streaks along the [110] direction of NbP (Figure 1d,e). The position of the additional streaks match with the reciprocal vector *q*_110_ = √2π/*a*, which corresponds to a real‐space lattice distance of √2a. This can be understood in terms of adsorption of P‐atoms in a square lattice of spacing √2a (see red dots in the sketch next to Figure 1e), which is in good agreement with the atomically resolved lattice observed by STM.

**Figure 2:**
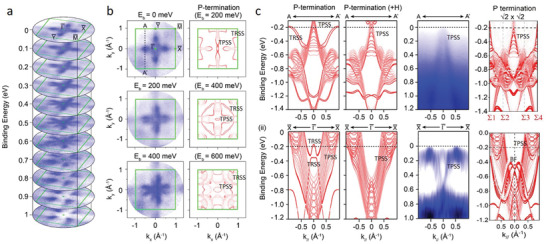
Suppression of trivial surface states in NbP via phosphorous surface reconstruction and saturation of dangling bonds. a) Momentum‐resolved photoemission spectroscopy in the *k_x_*–*k_y_* plane of a 15 nm‐thick NbP film, and its evolution with binding energy summarized in a stack plot. b) Constant‐energy *k_x_*–*k_y_* contour plots at representative energies, compared with calculations considering a P surface termination. Spoon‐like (topological) and bowtie features (trivial) are labelled with TPSS and TRSS, respectively. An energy shift of Δ*E* = 0.2 eV is considered for the calculated spectra to match with the experiment. c) Energy dispersion cuts along the A–A’ (top) and Γ**‐**X (bottom) directions, the former corresponding to the cut across the Weyl point projection (*k_x_* = 0.56 Å^–1^). Calculations of the P‐terminated surface states with and without dangling bonds reveal the origin of trivial surface bands (TRSS). Only topological surface states (TPSS) are found in the experiments, matching well with the scenario of dangling bond saturation. The calculations of the √2 × √2 surface reconstruction (rightmost panels) also feature the observed topological bands and linear dispersion along A–A'.

In order to investigate the effect of the surface reconstruction on the topological properties of NbP, photoemission spectra have been taken using an in‐house‐constructed momentum microscope^[^ ^28^ ^]^ with a He–I source (see Experimental Section for details). **Figure** **2** summarizes the overall electronic structure of a 15 nm‐thick NbP thin film measured at 100 K, including momentum distribution at the Fermi‐energy, energy dispersion along high‐symmetry cuts, and evolution of the *k_x_*–*k_y_* contours at different binding energies, together with ab initio calculations using the experimentally obtained lattice parameters. At the Fermi‐energy four hole pockets with an elliptical shape directed toward the X and Y symmetry points can be identified. With increasing binding energy, these pockets open up and evolve into a flower‐like shape (0.4–0.7 eV) and finally to four diagonal pockets with no density of states around the Γ point (0.8–1.0 eV), as visualized in the energy‐dependent stack plot in Figure 2a. Looking at the details of the *k_x_*–*k_y_* distribution and its comparison with the calculation of the termination‐dependent surface states (Figure 2b), it becomes evident that the elliptical band features along Γ–X and Γ–Y are characteristic of a P‐terminated surface. Interestingly, the size and shape of the measured elliptical (also called spoon‐like) features match with the calculated spectra (and with the bulk crystal data in refs. ^[^ ^9^, ^10^, ^29^ ^]^ considering a −0.2 eV Fermi‐level shift, as shown in Figure 2b.

Besides the energy shift, there is another noteworthy difference between the photoemission data of our thin films and bulk crystals (and the calculated band structure thereof) visible in the *k_x_*–*k_y_* constant energy contours. While the spoon‐like features (labelled as TPSS in Figure 2b) can be identified in both experiment and calculations, there is a complete absence of the bowtie‐like surface states (labelled as TRSS) around the X and Y points. In this regard, Liu et al.^[^ ^10^ ^]^ performed a very rigorous study of the effect of surface cleaving on the measured photoemission data of bulk crystals, finding out that the bowtie features were strongly affected by the cleavage site and hence the dangling bond configuration of the surface. These bowtie‐like surface states have been therefore classified as trivial surface states (TRSS),^[^ ^10^ ^]^ in contrast to the spoon‐like surface states which arise from the projection of the topological bulk bands (TPSS). In our as‐grown thin film surfaces, we expect a negligible amount of dangling bonds, as the surface undergoes a reconstruction under the P‐rich atmosphere, as confirmed by RHEED and STM (Figure 1). In turn, by producing an ordered surface based on a P‐termination in the MBE process, the trivial surface states originating from dangling bonds are completely suppressed, such that all the observed surface bands are of topological origin (TPSS). A consistent picture is visualized in Figure 2b, where the evolution of the topological bands only (without trivial surface bands) is observed at different binding energies. In order to confirm the dangling bond origin of the trivial surface states, ab initio calculations with H‐saturated dangling bonds on a P‐terminated surface have been carried out. Figure 2c compares the surface state calculations for an unsaturated and saturated surface along two relevant energy dispersion cuts, together with the obtained experimental data. In both dispersion cuts (A→A’ and Γ→X), it is clear how the sharp trivial surface bands (TRSS) disappear upon saturation of dangling bonds, matching well with the measured spectra (considering a 0.2 eV Fermi‐level shift). The A→A’ cut (Figure 2c‐i) is expected to cross the location of one pair of Weyl‐points in NbP (*k_x_* = 0.56 Å^–1^, *E*_b_ = −0.026 eV) and thus is used to visualize the surface Fermi arcs.^[^ ^9^, ^10^ ^]^ In our case, the A→A’ cut displays a clean, single linear band which characterizes the dispersion of the topological spoon‐like features (TPSS), whereas the W‐shaped bands (trivial‐ TRSS) are fully absent. Consistent results are found for the Γ→X dispersion cut (Figure 2c‐ii) as well. In the latter case, a second derivative plot was needed to visualize the V‐shape bands that evolve from the spoon‐like features (note that the topologically derived states feature a much lower intensity compared to the trivial surface states^[^ ^9^, ^10^, ^29^ ^]^). In a second calculation, we go beyond the effect of mere dangling bond saturation, and evaluate the role of a P‐terminated √2 × √2 reconstructed surface. As shown in the rightmost panel of Figure 2c, the energy dispersion cuts show again only topological bands with similar features as with the (1 × 1) H‐saturated surface, showing that this particular reconstruction does not affect the topological features and Fermi‐level shifts. Additional linear bands are observed close to the Σ1 and Σ4 points of the √2 × √2 supercell which correspond to a second pair of Weyl points (for details see Figure S2 in the Supporting Information) and along the Γ→X due to band folding, which are not relevant to the experiment. It is worth noting that only the direction Σ2–Σ3 of the supercell is strictly overlapping with the AA´cut of the primitive cell, such that just TPSS states are observed in the measurements. While it is clear that the topological surface bands are the dominant features in our P‐terminated (√2 × √2) NbP films, the Fermi‐arcs cannot be distinguished since *E*_F_ is 0.15 eV away from the Weyl points, and secondly, due the intrinsically short separation of the Weyl points in momentum space (Δ*k* < 0.05 Å^–1^), a challenge even for high‐resolution synchrotron ARPES.^[^ ^9^, ^10^, ^29^ ^]^

**Figure 3:**
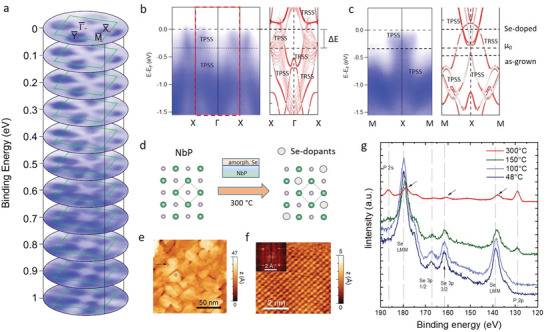
Large Fermi‐level shift in WSM thin films induced by surface doping. a) Stack plot of constant energy contours of a NbP film doped with Se, where the four electronic pockets at lower binding energies (close to *E*_F_) evolve from spoon‐like (topological) surface bands. b,c) Energy dispersion cuts along the Γ–X and M–X directions, together with band structure calculations including topological (TPSS) and trivial (TRSS) surface states. Besides the suppression of trivial surface states, as in the case of the as‐grown NbP films, a large energy shift of 0.5 eV with respect to the undoped NbP (and 0.3 eV with respect to the intrinsic chemical potential *µ*_0_) is achieved without distorting the overall bandstructure features. d) Schematic description of the Se‐doping process. e,f) Scanning tunneling microscopy images of a NbP surface after Se‐doping, featuring a square‐shaped grain topography and a 1 × 1 square lattice, showing that the surface structure of NbP is not modified. g) Temperature‐dependent X‐ray photoemission data during the Se‐doping process. At 300 °C, both P and Se (marked by arrows) are visible on the surface.

Moreover, taking advantage of MBE growth to achieve a controlled surface chemical doping, Se‐atoms have been employed to shift up the Fermi‐level of the as‐grown, hole‐doped NbP films (see Experimental Section for details). Selenium was chosen as dopant since it has a larger electronegativity than phosphorous and could eventually substitute P while retaining the host NbP crystal structure. A full replacement of P^–3^ by Se^–2^ atoms in the surface unit cell would lead to 1 extra electron (+1 eV shift). **Figure** **3**a–c summarizes the photoemission results after Se‐doping. The Fermi surface (Figure 3a) is evidently different compared to the as‐grown film, with much bigger elliptical pockets around the X and Y points, but retaining the four‐fold symmetry. There is a substantial energy shift (+0.5 eV with respect to the as‐grown film) upon Se‐doping, which becomes evident by comparing the dispersion cuts along high‐symmetry directions with the band structure calculations of the undoped NbP (Figure 3b,c). Most importantly, the overall shape of the bands belonging to NbP are not drastically affected by the Se‐doping process: the dominant effect is a chemical potential shift. These results point to a successful substitution of Se‐atoms on P‐sites without a structural change at the surface, although it induces a certain amount of disorder which manifests in the broadening of the electronic bands with respect to the undoped case. The resulting Fermi‐energy upshift by 0.5 eV (n‐type doping), allows the mapping of the electronic states of the hitherto unexplored energy region above the Weyl points. For instance, in the electron‐doped region, asymmetric electronic bands around the X and Y points are observed (Figure 3a), which, according to the calculations, evolve from the spoon‐like, topological features close to *E*_F_ (Figure 3b, indexed as TPSS bands for consistency). The dispersion cuts along M–X (Figure 3c), where a set of Weyl points are expected,^[^ ^9^, ^10^, ^29^ ^]^ are governed by the characteristic hourglass‐shaped bands. Consistent with the scenario observed in the as‐grown films, the absence of the trivial surface states (parabolic hole bands in the calculations, labelled with TRSS in Figure 3c) is evident, indicating that dangling bonds have been also saturated during the selenium surface doping process. However, an increased band broadening is observed in the Se‐doped samples, which might be produced by a small amount of disorder in the distribution of substitutional Se‐dopants on the surface, and a certain degree of band renormalization due to Se‐incorporation. Although individual bands cannot be well resolved, the overall behavior can be well mapped with the present data, yielding a 0.3 eV energy shift compared to the intrinsic chemical potential *µ*_0_, 0.35 eV to the bulk crystals^[^ ^9^, ^10^, ^29^ ^]^ and 0.5 eV with respect to the as‐grown films. We could reproduce this substantial energy shift by carrying out the Se‐doping experiment in a TaP film (Figure S3 in the Supporting Information), showing that the employed surface doping method is reproducible and works for other members of the monopnictide Weyl semimetal family. Figure 3d shows schematically the substitutional doping process on the NbP surface lattice, based on scanning tunneling microscopy images of the NbP surface after doping (Figure 3e,f) which display an atom‐resolved topography characteristic of the 1 × 1 surface lattice of pristine NbP. In order to verify the inclusion of Se‐atoms on the surface, core‐level spectra have been taken at various stages of the doping process (deposition of an amorphous Se‐layer onto the NbP film and a subsequent heating procedure to 300 °C to achieve the incorporation of Se in the lattice). Figure 3g shows the results of the in situ temperature‐dependent X‐ray photoemission study during the doping process. At 100 °C, the Se layer fully covers the surface (no P peaks), whereas from 150 °C to 300 °C the excess Se desorbs and a certain amount of it is incorporated into the surface, as the intensity of the P atoms gradually increases while that of Se decreases. Although the concentration of Se‐dopants cannot be determined accurately, the presence of both P and Se species at the surface after the doping process is verified. Combined with the structural data obtained by STM, this observation points to a successful Se incorporation in the NbP lattice as sketched in Figure 3d. Summarizing, the most remarkable result of the employed doping strategy is that a chemical potential shift has been concomitantly achieved with the suppression of trivial surface states, demonstrating Fermi‐level engineering of purely topological bands.

## 3 Conclusion

We have demonstrated efficient routes to modify the electronic structure of Weyl semimetals by thin film growth engineering. By preparing a homogeneous phosphorous termination (manifested in a √2 × √2 surface reconstruction) that leads to a saturation of dangling bonds, the trivial surface states are entirely suppressed with respect to the topological ones, establishing an excellent platform to visualize, manipulate and make exclusive use of topological surface states. Using a careful doping approach, a route to substantially modify the Fermi‐level of WSMs – thereby retaining the topological properties of the host crystal‐ is successfully demonstrated. These findings are a major progress with regard to Fermi‐level engineering of purely topological bands in Weyl semimetals, and a key step toward the achievement of Fermi‐level tunable topological heterostructures and functional Weyltronic devices.

## 4 Experimental Section

### Molecular‐Beam Epitaxy

MgO (100) substrates were chemically cleaned as described in ref. ^[^ ^25^ ^]^ prior to NbP film growth. Nb rods are evaporated via electron‐beam heating and P species are thermally evaporated from a GaP compound effusion cell in a custom‐made UHV chamber (*p*_base_ = 1 × 10^–10^ mbar). A cross‐beam mass spectrometer (XBS Hiden) is used to calibrate the atomic fluxes, and the substrate temperature is controlled by radiation heating. The NbP layer is grown under P‐rich conditions (Nb:P flux 1:20), a moderate substrate temperature (300–400 °C) and a slow rate (4 nm h^−1^), controlled by the Nb‐flux. After concluding the growth process, the sample is cooled down very slowly (10 °C min^−1^) to room‐temperature under P‐atmosphere (p = 1 × 10^–8^ mbar), to ensure a homogeneous P‐termination at the surface.

### Surface Doping

An amorphous Se‐layer was deposited at room‐temperature using a standard effusion cell onto a freshly grown NbP epitaxial film, until the surface acquires a reddish color, a similar approach used to prepare Se‐capping layers. The Se‐capped NbP was then rapidly transferred to the momentum microscope, with a short exposure to air. Before taking the photoemission data shown in Figure 3, the sample was annealed to 300 °C for 1 h in ultrahigh‐vacuum, allowing the reaction and substitutional inclusion of Se‐atoms at the Se/NbP interface, and promoting the desorption of residual Selenium far from the interface. After the surface doping process, the sample has been investigated via in situ STM, confirming the preservation of the NbP surface structure.

### In Situ Characterization

The crystallinity of the films is monitored in situ by reflection high‐energy electron diffraction (RHEED), using a 15 kV electron beam. The layers are further characterized by in situ tools (XPS, LEED, STM and momentum‐resolved photoemission) with the help of a vacuum suitcase transfer system (Ferrovac, *p*_base_ < 1 × 10^–10^ mbar). Core‐level X‐ray photoelectron spectra (XPS) before and after Se‐doping was taken at room‐temperature using Al Kα radiation and a hemispherical analyzer. The STM experiments were performed on an Omicron VT‐STM‐XT system operated at room temperature and under a base pressure of 2 × 10^–11^ mbar. The mechanically sharpened Pt/Ir tips were treated and checked on Au(111) surface before measurements, and the topography images were acquired at room‐temperature. Momentum‐resolved photoemission spectra was acquired using an in‐house designed momentum microscope,^[^ ^28^ ^]^ at 100 K and using He I radiation (21.2 eV). The NbP film with P‐reconstruction was transferred to the Momentum Microscope system by using the vacuum suitcase.

### Ab Initio Calculations

The electronic structure calculations are performed within the density functional theory as implemented in the Vienna Ab initio Simulation Package (VASP).^[^ ^30^ ^]^ The exchange‐correlation energy is treated within the Perdew–Burke–Ernzerhof (PBE) parametrization^[^ ^31^ ^]^ of the generalized gradient approximation throughout. The kinetic energy cutoff for the plane wave basis is 300 eV and a k‐mesh of 500 × 500 × 1 and 300 × 300 × 1 is adopted for the Brillouin zone integration for the 1 × 1 pristine surface structure and for the √2 × √2 reconstructed surface, respectively. A six‐unit‐cell thick slab model is constructed by cutting NbP along the (001) plane of the conventional cell and the in‐plane cell size is a 1 × 1 conventional cell. The internal coordinates of the atoms in the surface unit cell are fully relaxed. Spin‐orbit coupling is considered in the electronic structure calculations. All the surface band structures and fermi surfaces are projected onto the atoms in the surface unit cell of the P‐, P–H and Nb‐termination, accordingly.

## Conflict of Interest

The authors declare no conflict of interest.

## Author Contributions

A.B‐P. developed the film growth and surface doping strategy and performed RHEED characterization. D.L. performed the momentum‐resolved photoemission measurements and data analysis. H.T. calculated the band structure and termination‐dependent surface states. A.K.P assisted with the thin film growth. K.C. carried out the STM measurements and J.Z. the temperature‐dependent XPS measurements. A.B‐P. wrote the manuscript with input of all authors. S.S.P.P. supervised the entire project.

## Supporting information

Supporting Information

## Data Availability

The data that support the findings of this study are available from the corresponding author upon reasonable request.
