# Supplementary material for: Large Fermi‐Energy Shift and Suppression of Trivial Surface States in NbP Weyl Semimetal Thin Films
Source: Adv Mater. 2021 May 4;33(21):2008634. doi: 10.1002/adma.202008634 (PMC11469305; doi:10.1002/adma.202008634)
Supplement: Supplementary file 1 — Supporting Information [file ADMA-33-2008634-s001.pdf]

© 2021 Wiley-VCH GmbH

ADVANCED MATERIALS

Supporting Information

for *Adv. Mater.,* DOI: 10.1002/adma.202008634

Large Fermi-Energy Shift and Suppression of Trivial Surface States in NbP Weyl Semimetal Thin Films

Amilcar Bedoya-Pinto, Defa Liu, Hengxin Tan, Avanindra Kumar Pandeya, Kai Chang, Jibo Zhang, and Stuart S. P. Parkin**

Supporting Information

DOI: 10.1002/((please add manuscript number))

Article type: Communication

Large Fermi-Energy Shift and Suppression of Trivial Surface States in NbP Weyl Semimetal Thin Films

Amilcar Bedoya-Pinto[§], Defa Liu[§], Hengxin Tan, Avanindra Kumar Pandeya, Kai Chang, Jibo Zhang and Stuart S.P. Parkin**

Dr. A. Bedoya-Pinto, Dr. Defa Liu, Dr. Hengxin Tan, Avanindra Kumar Pandeya, Dr. Kai Chang, Jibo Zhang and Prof. Dr. Stuart. S.P. Parkin
Max Planck-Institute of Microstructure Physics,
Weinberg 2, 06120 Halle (Saale), Germany

*E-mail: abedoya@mpi-halle.mpg.de,
stuart.parkin@mpi-halle.mpg.de

§ These authors contributed equally to this work

Keywords: Weyl Semimetal thin films, topological surface states, Fermi arcs, Fermi-level engineering

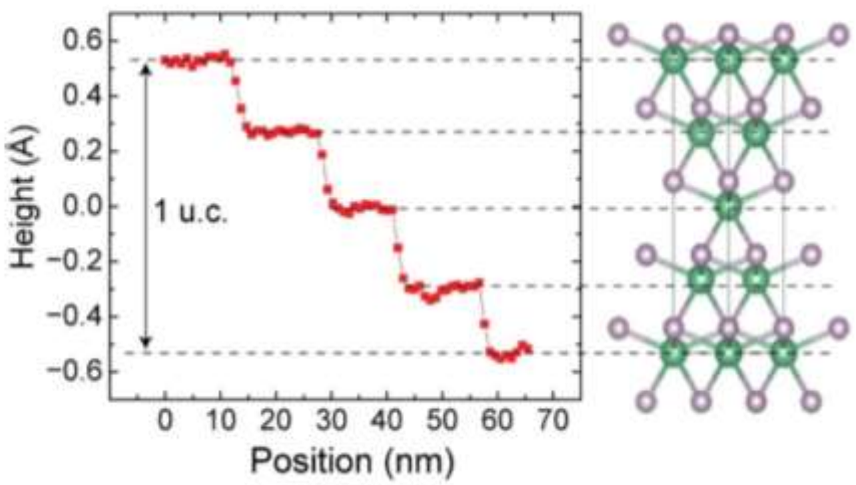

Fig. S1: Height profile across the surface of NbP, taken from an overview scanning tunneling microscopy image (Figure 1a – marked with an arrow). Atomically-flat terraces, corresponding to each Nb-P layer (2.8 Å) of the NbP structure, can be well distinguished. A NbP unit cell in (001) direction, composed of 4 Nb-P individual layers, is drawn as guide to the eye.

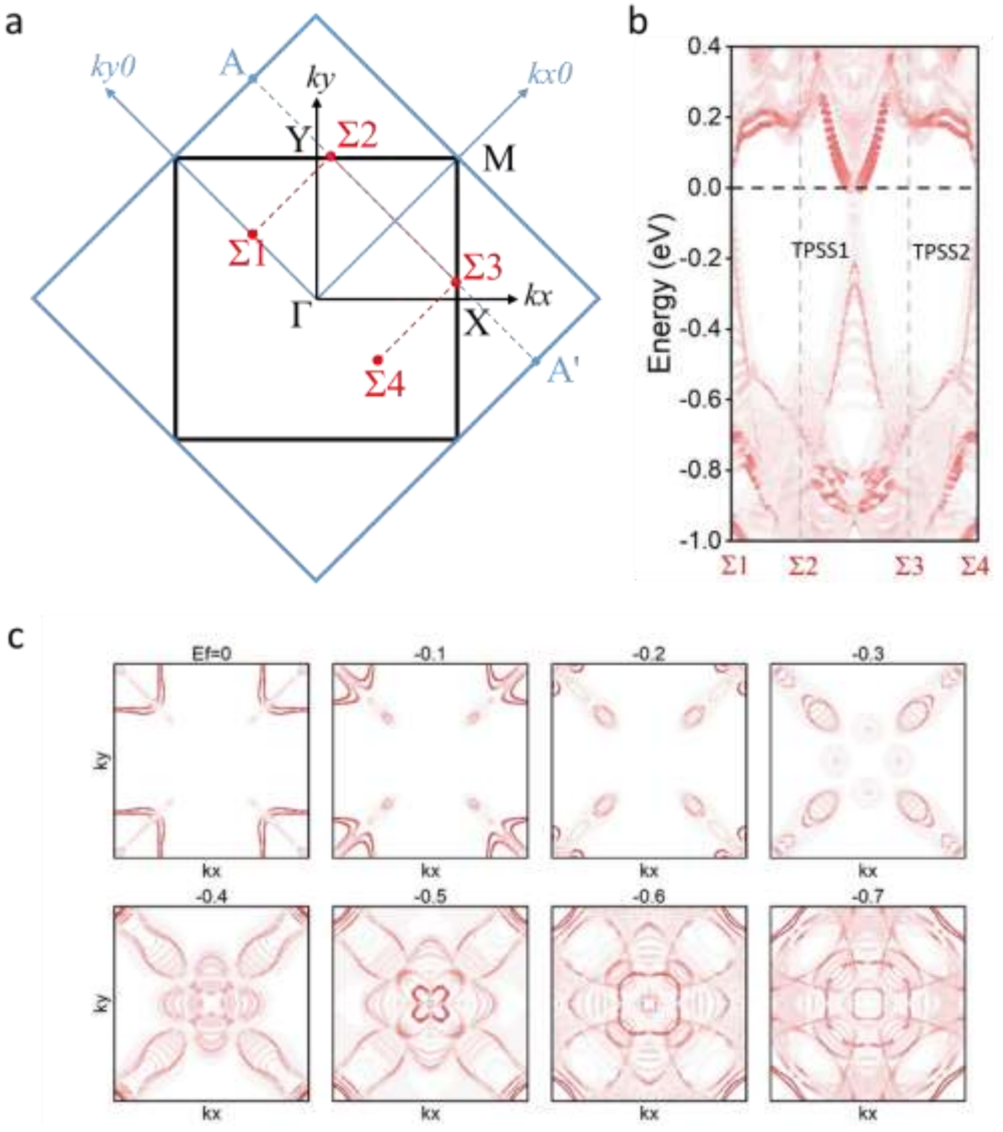

Fig. S2: Band structure of the P (√2 × √2) reconstructed NbP surface. a) The relationship between the 1st Brillouin Zones for the primitive cell (blue) and √2 × √2 supercell (black). The AA' line in the Brillouin Zone of the primitive cell corresponds to a line cut across a pair of Weyl points. b) Surface band structure for the supercell along the *k* paths as shown in a), showing topological surface states (TPSS1) at the positions where the Weyl points (along AA´) are expected. Note that the Σ1 (Σ4) is close to the other pair of Weyl points on k_{y0} axis of the primitive cell Brillouin Zone, which results in the states near the Fermi-level Σ1 (Σ4) and a linear dispersion (TPSS2). c) Surface-projected constant-energy contours of the √2 × √2 supercell at different binding energies (in eV). In both b) and c), the Fermi level is set as the energy zero.

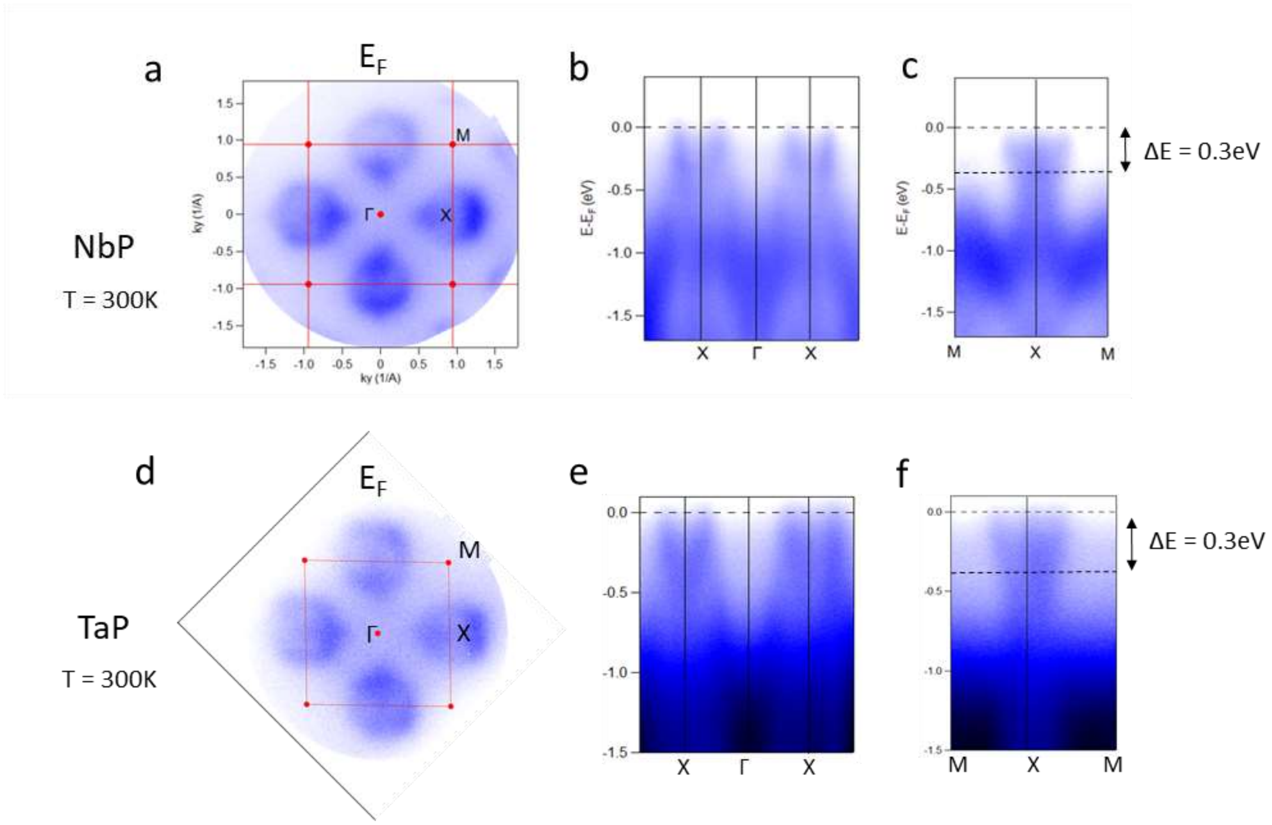

Figure S3: Electronic structure upon Se-doping in NbP (a-c) and TaP (b-d) thin films. Constant-energy contours at the Fermi-energy show very similar electronic pockets following four-fold symmetry for NbP (a) and TaP (d) thin film surfaces. The dispersion cuts along Γ→X and M→X directions evidence a Fermi-level shift of around 0.3eV with respect to the intrinsic chemical potential both for NbP (b,c) and TaP (e,f). These results highlight the reproducibility of the Se-doping strategy to account for Fermi-level engineering on the Weyl monopnictide family.
